# Blood-Glucose-Lowering Effect of *Coptidis Rhizoma* Extracts From Different Origins *via* Gut Microbiota Modulation in *db/db* Mice

**DOI:** 10.3389/fphar.2021.684358

**Published:** 2021-06-15

**Authors:** Yuanfeng Lyu, Lin Lin, Yuning Xie, Dan Li, Min Xiao, Yufeng Zhang, Stanley Chun Kai Cheung, Pang Chui Shaw, Xiao Yang, Paul Kay Sheung Chan, Alice Pik Shan Kong, Zhong Zuo

**Affiliations:** ^1^ School of Pharmacy, Faculty of Medicine, The Chinese University of Hong Kong, Shatin, China; ^2^ Department of Microbiology, Faculty of Medicine, The Chinese University of Hong Kong, Shatin, China; ^3^ School of Life Sciences and Li Dak Sum Yip Yio Chin R&D Centre for Chinese Medicine, The Chinese University of Hong Kong, Shatin, China; ^4^ Division of Endocrinology, Department of Medicine and Therapeutics, Faculty of Medicine, The Chinese University of Hong Kong, Shatin, China

**Keywords:** *Coptidis rhizoma* extracts, gastrointestinal bacteria, gut microbiota, db/db mice, diabetes

## Abstract

**Background:** *Coptidis rhizoma* extracts (CREs) have been used widely for their anti-diabetic and anti-microbial activities, and berberine/jatrorrhizine/coptisine/palmatine are the primary bioactive components. Although guidelines have adopted content analyses of these components as a quality control method for CREs, it is difficult to differentiate the CREs from different sources using this method because of the lack of indications for their related pharmacological activities.

**Purpose:** To explore the effect of CREs (CREA/CREB/CREC) with different compositions of major components on the gut microbiota and blood glucose levels in d*b/db* mice.

**Methods:** Degradation of berberine/jatrorrhizine/coptisine/palmatine from CREA/CREB/CREC in rat/mouse intestinal contents and their impact on nine common gastrointestinal bacteria were investigated. In addition, the effects of oral administration of CREA/CREB/CREC for 2 weeks on the gut microbiota and blood glucose levels in d*b/db* mice were monitored *via* insulin/glucose tolerance test (ITT/GTT), insulin concentration, homeostatic model assessment of insulin resistance and fecal 16S rRNA sequencing.

**Results and Conclusion:** The total amount of berberine/jatrorrhizine/coptisine/palmatine was highest in CREA. *Clostridium perfringens* was strongly inhibited by all three CREs, with CREA demonstrating the most significant inhibitory effects on minimum inhibitory concentration, time-kill kinetics, and ATP production. In d*b/db* mice, CREA resulted in the most significant decrease in ITT/GTT and depicted different changes in the microbiota from CREB/CREC. Thus, CREs with different compositions of berberine/jatrorrhizine/coptisine/palmatine differed in terms of time-kill kinetics and ATP production assays on *C. perfringens*. CREA revealed the potent bacterial inhibitory effects and glucose-lowering activity.

## Introduction

*Coptidis rhizoma* (CR), the dry root of Coptis chinensis Franch., has been used clinically in patients with diabetes and gastrointestinal (GI) diseases throughout the long history of traditional Chinese medicine (Chinese Pharmacopoeia Commission, 2015). Its herbal extracts have been widely reported and recognized for their pharmacological roles in a variety of therapeutic applications, including anti-diabetic (improving glucose uptake and insulin resistance) and anti-microbial activities (anti-bacteria, anti-virus, anti-fungi) (Hwang et al., 2003; Choi et al., 2007; Yin et al., 2008; Feng et al., 2011; Chang et al., 2014). To date, more than 100 chemical components have been identified in CR, including alkaloids, lignans, simple phenylpropanoids, and flavonoids. Among these, alkaloids are the primary bioactive ingredients, and play vital roles in the pharmacological effects of CR (Wang et al., 2019).

Berberine (BBR) is the major active marker isolated from CR; it is a natural isoquinoline alkaloid, which has been shown to exhibit activity against inflammation and hypercholesterolemia (Kuo et al., 2004; Brusq et al., 2006). In addition to BBR, CR contains three key chemical constituents that are considered the main active components: jatrorrhizine (JAT), coptisine (COP), and palmatine (PAL) (Wang L. et al., 2014; Yang et al., 2018). According to the Hong Kong Chinese Materia Medica Standards (HKCMMS), the contents of BBR hydrochloride and PAL hydrochloride should be no less than 4.1 and 0.3%, respectively, in dried CR decoction slices [Hong Kong Chinese Materia Medica Standards (HKCMMS), 2018]. Furthermore, BBR should be no less than 5.0%, according to the 2015 edition of Pharmacopeia of the People’s Republic of China (Chinese Pharmacopoeia Commission, 2015).

As indicated by previous findings, BBR demonstrates glucose lowering effect with improved insulin resistance in different diabetic-related animal models, including high fat die-fed C57BL/6J mice and streptozotocin/alloxan-induced rats (Tang et al., 2006; Zhang et al., 2008; Kong W. J. et al., 2009; Chen et al., 2010; Liu et al., 2010; Shen et al., 2012). Moreover, Ma et al. revealed that after COP/PAL/JAT at 225 mg/kg orally administered to KK-Ay mice for 40 days, the fasting blood glucose and glucose tolerance levels of mice were significantly ameliorated and improved (Ma et al., 2015). In addition, studies have suggested that BBR may be used to treat patients with type 2 diabetes (T2D) by modulating and altering the structure and composition of the microbiome (Liu et al., 2010). Due to its poor oral bioavailability (less than 1%), the rate of BBR absorption in the GI tract is no more than 10% (Liu et al., 2010; Ma et al., 2013). Studies have reported that approximately 60% of BBR remained in the intestinal tract of Sprague-Dawley rats, and only 10–25% of BBR was degraded in human intestinal bacteria/human fecalase after 24 h of *in vitro* incubation (Lyu et al., 2019). These findings suggest that BBR can inhibit certain Gram-positive (G^+^) bacteria, including *Staphylococcus aureus* (*S. aureus*) and *Streptococcus agalactiae* (Fan et al., 2008; Wang L. et al., 2014; Peng et al., 2015). The growth of Gram-negative (G^-^) bacteria, such as *Shigella dysenteriae* and *Actinobacillus pleuropneumoniae,* can also be suppressed by BBR (Kong et al., 2010; Kang et al., 2015). Studies have shown that CR extracts (CREs) exert strong antibacterial effects against the growth of *Streptococcus mutans* (Choi et al., 2007). *S. aureus* and *Salmonella typhimurium* growth was inhibited by CREs with a minimum inhibitory concentration (MIC) of 77.8 μg/ml and a minimum bactericidal concentration of 12.5 mg/ml (Feng et al., 2011; Chang et al., 2014). Moreover, BBR was shown to suppress *Escherichia coli* (G^-^, *E. coli*) growth, and the antibacterial activities of the other three main active alkaloids in CR have demonstrated inhibitory effects on *E. coli*, in the following order: BBR > COP > PAL > JAT (Kong W. et al., 2009; Boberek et al., 2010). In addition, CR has been demonstrated to significantly ameliorate the composition of gut microbiota and improve glycolipid metabolism in diabetic rats (Xiao et al., 2020). The abundance and diversity of intestinal flora can also be reversed and modulated by CR alkaloids in obese/hyperlipidemic mice (He et al., 2017).

Considering the potential roles of BBR, JAT, COP, and PAL in the effect of CREs on blood glucose levels and gut microbiota, it is hypothesized that CREs with different compositions of these active components may differ in their impact and modulation on these outcomes. Thus, the current study aimed to 1) investigate the effects of gut microbiota from Sprague-Dawley rats and C57BL/6 mice on the major active components from different CREs; 2) evaluate the impacts of major active ingredients and their different compositions on nine common GI bacteria present in rats and humans; and 3) evaluate the impacts of different CREs on blood glucose levels and bacterial profiles of diabetic d*b/db* mice.

## Materials and Methods

### Herbal Materials

Three dried roots of CR (CRA/CRB/CRC) from different origins were purchased from the Bei Sha Yao Cai manufacturer (Foshan, Guangdong, P. R. China), among which CRA was identified as the genuine medicinal material. The voucher specimens for the three herbs (CRA [T5047A]; CRB [T5047B]; CRC [T5048]) were kept in the Li Dak Sum Yip Yio Chin R&D Center for Chinese Medicine at The Chinese University of Hong Kong.

### Chemicals and Reagents

BBR chloride (purity ≥97%) was purchased from Sigma-Aldrich. Co., Milwaukee, WI, United States. JAT hydrochloride, COP chloride, PAL hydrochloride (purity ≥98%), and caffeine (CAF, internal standard [IS]; purity >98%) were purchased from Dalian Meilun Biotech Co., Ltd. (Dalian, Liaoning, P. R. China). Acetonitrile (ACN), methanol (MeOH), and dimethyl sulfoxide (DMSO) (RCI Labscan Limited, Bangkok, Thailand) were used for high-performance liquid chromatography (HPLC). Distilled and deionized water were supplied by a Millipore water purification system (Milford, MA, United States).

### Preparation of Aqueous Extract of CRA/CRB/CRC (CREA/CREB/CREC) and Content Analyses of BBR/JAT/COP/PAL

Water extraction procedure was performed as described in the previous literature (Cui et al., 2018). In brief, dry herb pieces of CRA/CRB/CRC (0.3 kg) were immersed, extracted with boiling distilled water (1:10, w/v) twice for 2 h under reflux, passed through a 0.45 μm filter, and evaporated to powder to obtain the corresponding extracts of CREA/CREB/CREC. HPLC fingerprints of CREA/CREB/CREC, shown in Supplementary Figure S1, were obtained using Waters 996A HPLC/UV (Milford, MA, United States). BBR/JAT/COP/PAL were separated *via* Agilent Eclipse XDB-C18 (4.6*250 mm, 5 μm) column (Agilent Technologies, Santa Clara, CA, United States) by gradient elution with a mixture of 0.75% formic acid and 5 mM ammonium acetate as the aqueous component of the mobile phase and ACN (time [min], percentage of ACN: 0–5, 7%; 5–30, 7–60%; 30–32, 60–70%; 32–37, 70–7%; 37–47, 7%) at a flow rate of 0.8 ml/min, with UV detection at 346 nm wavelength.

LC-MS/MS, performed *via* Agilent 6430 triple quadrupole liquid chromatography/tandem mass spectrometry (Agilent Technologies, Santa Clara, CA, United States), was used for the content analyses of BBR/JAT/COP/PAL in CREA/CREB/CREC. Liquid chromatographic separation of BBR, JAT, COP, PAL, and IS (CAF) was performed using an Agilent Extend-C18 (2.1 × 150 mm, 5 μm) analytical column (Agilent Technologies, Santa Clara, CA, United States) with a mobile phase consisting of 0.1% formic acid and ACN (70:30, v/v) at a flow rate of 0.5 ml/min. Quantification was determined by multiple reaction monitoring (MRM) for each compound as described previously (Han et al., 2011): BBR m/z 336.1→320.1, JAT m/z 338.2→322.8, COP m/z 320.1→292.1, PAL m/z 352.2→336, and IS (CAF) m/z 195.1→138.0.

### Effects of Rat/Mice Intestinal Contents on the Stability of Major Active Components in CREA/CREB/CREC

#### Animals

Male Sprague-Dawley rats (200–220 g), C57BL/6 mice (6 weeks old), and d*b/db* mice (6 weeks old, BKS.Cg-*Dock* ^*7m*^+/+*Lep* ^*db*^/J) were provided by the Laboratory Animal Services Center (Hong Kong SAR, P. R. China). All animals were acclimatized for 1 week prior to the experiments and housed under controlled conditions (25 ± 2°C temperature, 50 ± 5% humidity, 12 h light-dark cycle). All experimental procedures were approved by the Animal Ethics Committee (License No.: 18-022-MIS-5-C; 18-115-MIS-5-B) at The Chinese University of Hong Kong and the Department of Health of Hong Kong [License No.: (18-12) in DH/SHS/8/2/1 Pt.7; (18-223) in DH/SHS/8/2/1 Pt.9; (20-470) in DH/HT&A/8/2/1 Pt.8].

#### Preparation of Rat/Mice Intestinal Content Suspensions

Rat/mice intestinal contents were prepared as we described previously (Lyu et al., 2019). Briefly, intestinal contents from rats (4 g/rat) and mice (1 g/mouse) were homogenized under 4°C to obtain suspensions in 36 and 9 ml of 0.9% cold saline, respectively.

#### Incubation of CREA/CREB/CREC in Rat/Mice Intestinal Contents

CREA/CREB/CREC at 2 mg/ml (in purified water) was spiked into 950 μL rat/mouse intestinal content suspensions at a final concentration of 100 μg/ml. All mixtures were incubated at 37°C. Samples were collected before (0 h) and at 1, 2, 3, 6, and 24 h after the post-spike, and then frozen immediately at –80°C for further analyses.

#### Sample Treatment and LC-MS/MS Analyses

Each sample (50 μL) was added to 50 μL IS (CAF at 0.25 μg/ml in MeOH) and 200 μL organic solvents (ACN: MeOH = 1:1, v/v) to precipitate the proteins. After centrifugation at 18,600×g for 10 min, the supernatants were collected for LC-MS/MS analyses of BBR/JAT/COP/PAL, as described above. Calibration curves were prepared by spiking 25 μL BBR (0.39–12.5 μg/ml in MeOH)/JAT/COP/PAL (0.098–3.125 μg/ml in MeOH) standard solution in two-fold series dilutions, and 50 μL IS (0.25 μg/ml in MeOH) with 100 μL ACN into blank rat/mice intestinal content suspensions.

### Minimum Inhibitory Concentration of BBR/JAT/COP/PAL and CREA/CREB/CREC in Selected Bacteria

#### Preparation of Selected Bacteria

Nine common GI bacterial strains, including seven pathogenic bacteria (*E. coli*, *Klebsiella pneumoniae*, *Enterobacter cloacae*, *Enterococcus faecalis*, *S. aureus*, *Bacteroides fragilis*, and *C. Clostridium perfringens*), and two probiotic bacteria (*Bifidobacterium bifidum* and *Lactobacillus fermentum*) were included in the current study. All bacteria were cultured on Gifu Anaerobic Medium (GAM) agar plates and incubated anaerobically at 37°C for 24 h before MIC determination.

#### MIC Determination of BBR/JAT/COP/PAL, Their Physical Mixture, and CREA/CREB/CREC With Selected Bacteria

MIC, defined as the drug concentration at which no bacterial growth could be observed, was determined by the microbroth dilution assay in GAM broth according to the Clinical & Laboratory Standards Institute (CLSI) guidelines (Clinical and Laboratory Standards Institute (CLSI), 2020). Briefly, McFarland turbidity cells (100 μL) were inoculated into 15 ml GAM broth to obtain cell suspensions. Then, 50 μL of BBR/JAT/COP/PAL (5 mg/ml in DMSO) and CREA/CREB/CREC (50 mg/ml in DMSO containing BBR at 6,250 μM) stock solutions were added to 50 μL of the cell suspensions in a 96-well plate to obtain the final concentrations at 0.975–1,000 and 2.5320 μM (BBR concentrations in CREs) with two-fold serial dilutions, respectively. A mixture of BBR/JAT/COP/PAL standard compounds (Mixture A, Mixture B, and Mixture C) was prepared based on the amount of each component according to their contents in CREA/CREB/CREC. Then, CREA/CREB/CREC and their mixture (2.5320 μM) were incubated with the five selected bacteria (*S. aureus*, *B. fragilis*, *C. perfringens*, *B. bifidum*, and *L. fermentum*). Then, the 96-well plates were incubated anaerobically at 37°C for 24 h and then examined visually for bacterial growth.

#### Time-Kill Kinetics and ATP Production of CREA/CREB/CREC With *C. perfringens*

Time-kill kinetics were analyzed by incubating CREA/CREB/CREC with *C. perfringens* in GAM broth to demonstrate their antibacterial effects. Early log-phase cell cultures were used for the inoculation. In brief, 300 μL of 0.5 McFarland turbidity cells were inoculated into 30 ml GAM broth to obtain the starting inoculums and then aliquoted into individual tubes. CREA/CREB/CREC was added to 2 ml aliquots of *C. perfringens* suspension with the final concentrations corresponding to ¼×MIC (0.02 mg/ml), 1×MIC (0.08 mg/ml), 2×MIC (0.16 mg/ml), and 4×MIC (0.32 mg/ml). DMSO was added to the untreated control group. Culture tubes were incubated anaerobically at 37°C, and samples were collected at 0, 2, 4, and 6 h to determine total colony-forming units (CFUs) using the broth microdilution method. The number of colonies on the culture plate was counted after 24 h of anaerobic incubation. In addition, *in vitro* ATP production in cells was measured *via* the BacTiter-Glo™ Microbial Cell Viability Assay at 0, 2, 4, and 6 h after CREA/CREB/CREC incubation with *C. perfringens*, according to the manufacturer’s instructions (Promega, WI, United States). Briefly, 20 μL samples were mixed directly with 20 μL ATP assay reagent for 5 min at room temperature (25 ± 2°C). Luminescence was measured using a GloMax® 96 Microplate Luminometer (Promega, WI, United States).

### Effects of CREA/CREB/CREC on the Blood Glucose Levels and Gut Bacterial Profiles of d*b/db* Mice

#### Groups and Treatments

Based on the human equivalent doses (2–5 g/day/person) of CR decoction (Chinese Pharmacopoeia Commission, 2015) and scale factor between human and mice (Food and Drug Administration, 2005), each CRE at 200 mg/kg of were orally administered to d*b/db* mouse (6 weeks old, BKS.Cg-*Dock* ^*7m*^+/+*Lep* ^*db*^/J). Male d*b/db* and wild-type mice were randomly divided into five groups (*n* = 5/group): wild-type control (WT), diabetic control (db), CREA (CREA) at 200 mg/kg (containing BBR at 8.62 mg; JAT+COP+PAL at 8.10 mg), CREB (CREB) at 200 mg/kg (containing BBR at 8.62 mg; JAT+COP+PAL at 7.76 mg), and CREC (CREC) at 200 mg/kg (containing BBR at 8.08 mg; JAT+COP+PAL, at 6.4 mg). CREA, CREB, and CREC were suspended in 0.3% sodium carboxymethyl cellulose (CMC-Na). Mice in the WT and db groups received 0.3% CMC-Na, while the rest of the treatment groups received different CREs for 14 consecutive days *via* the intragastric route with body weight monitored.

#### Insulin Tolerance Test, Intraperitoneal Glucose Tolerance Test, Fasting Serum Insulin Concentration, and Homeostatic Model Assessment of Insulin Resistance

An insulin tolerance test (ITT) was performed to evaluate insulin sensitivity by monitoring the endogenous loss over time in response to an injection of human insulin. On day 13, mice were intraperitoneally (i.p.) injected with recombinant human insulin (0.75 IU/kg, after 4 h fasting). Blood drops were then collected *via* the tail vein to monitor blood glucose levels 0-, 30-, 60-, 90-, and 120-min post injection (Vinué and González-Navarro, 2015). Mice were fasted overnight after the last dose on day 14. On day 15, fasting serum was obtained from blood samples collected in tubes without anticoagulant, which was centrifuged at 4,300×g for 15 min. Serum samples were stored at −80°C for further analyses. Insulin concentrations were measured using a Millipore Rat/Mouse Insulin ELISA kit (Merck Millipore, MA, United States) (Borai et al., 2007). An intraperitoneal glucose tolerance test (ipGTT) was conducted on day 15 to measure the clearance of an injected glucose load by monitoring the blood glucose levels before and 30, 60, 90, and 120 min after i.p. injection of 1.0 g/kg glucose (Vinué and González-Navarro, 2015). Blood glucose levels were determined using a Contour® Plus glucometer (Bayer, Leverkusen, Germany). Based on the fasting insulin concentration and fasting blood glucose levels, homeostatic model assessment of insulin resistance (HOMA-IR) levels were calculated to quantify insulin resistance using the equation HOMA-IR = insulin concentration (mIU/L) * glucose level (mmol/L)/22.5, as described previously (Borai et al., 2007).

#### Fecal Sample Collection, Genomic DNA Extraction, and Microbial Amplicon 16S Ribosomal RNA (rRNA) V3-V4 Amplification, Sequencing, and Data Analyses

After 14 days of treatment (on day 15), fecal samples were collected from the bottom of the colon of each mouse, placed in frozen tubes, and stored at –80°C until further analyses. Bacterial genomic DNA was extracted from the stored fecal samples (180–220 g/fecal sample) using the QIAamp® PowerFecal® Pro DNA Kit according to the manufacturer’s instructions (QIAGEN, Hilden, Germany). DNA concentrations in the samples were determined by measuring the absorbance at 260/280 nm. Agarose gel (1%) electrophoresis was used to assess the quantity and integrity of the DNA (Supplementary Figure S2). The V3-V4 distinct regions of the 16S ribosomal RNA (rRNA) genes were amplified by polymerase chain reaction (PCR) using the primers 341 F (CCTAYGGGRBGCASCAG) and 806 R (GGACTACNNGGGTATCTAAT). PCR amplification was performed with 15 µL of Phusion® High-Fidelity PCR Master Mix (New England Biolabs, MA, United States), 2 µM of forward/reverse primers, and 10 ng template DNA under the following conditions: initial denaturation at 98°C for 1 min followed by 30 cycles of denaturation at 98°C for 10 s, annealing at 50°C for 30 s, and elongation at 72°C for 30 s. Final extension was performed at 72°C for 5 min. PCR products were mixed with 1× loading buffer, subjected to agarose gel (2%) electrophoresis, and purified using the QIAquick® Gel Extraction Kit according to the manufacturer’s instructions (QIAGEN, Hilden, Germany). Sequencing libraries were generated using the Illumina platform. Library quality was assessed using a Qubit® 2.0 Fluorometer (Thermo Scientific, MA, United States) and Agilent Bioanalyzer 2100 system (Agilent Technologies, Santa Clara, CA, United States). Paired-end reads were merged using FLASH V1.2.7 software. Quality filtering was performed *via* the QIIME V1.7.0 quality-controlled process (http://qiime.org/index.html). Effective tags were obtained using the Gold database *via* the UCHIME algorithm. Sequence analyses were performed using Uparse V7.0.1001 software (http://drive5.com/uparse/). Operational taxonomic units (OTUs) with over 97% sequence similarity were assigned and screened for annotation. The GreenGene Database (http://greengenes.lbl.gov/cgi-bin/nph-index.cgi) was used to obtain taxonomic information for the representative sequences. The alpha diversity index was based on the Mothur V1.30.1 software. The beta diversity index was determined using the QIIME V1.7.0 software.

### Statistical Analyses

Statistical analyses for normal distribution were conducted using one-way analysis of variance (ANOVA). Wilcoxon signed-rank tests were performed with paired comparisons between groups. Analyses were performed using GraphPad Prism software (version 9.00, San Diego, United States). In addition, Pearson correlation analyses (data with normal distribution) using R software package (version 4.1.1) were utilized to identify the underlying correlation analyses of gut microbiota alteration and glucose lowering activity among different CRE treatments in d*b/db* mice. Data are presented as mean ± standard deviation (SD). Statistical significance was set at *p* < 0.05.

## Results

### Analyses of CREA/CREB/CREC Contents in Decoction Slices

The concentrations of BBR, JAT, COP, and JAT in different CREs were detected using LC-MS/MS analytical methods. Marker content in decoction slices (w/w) was calculated as the product of yield in water extraction (CREA, 25.76%; CREB, 24.68%; CREC, 23.70%) and the concentration of each marker in CREA/CREB/CREC. As shown in Table 1, among the four active ingredients, BBR was the most abundant in CREA, CREB, and CREC, at 4.31 ± 0.30, 4.31 ± 0.31, and 4.04 ± 0.39% (w/w), respectively. The amounts of JAT and COP in CREC were significantly lower than that in CREA and CREB. In addition, the total amount of JAT, COP, and PAL in CREA (4.05 ± 0.06%, w/w) was the highest among the three CREs, with a significant difference compared to that from CREB and CREC (*p* < 0.05).

**TABLE 1:** Content analyses of major active compounds (berberine/jatrorrhizine/coptisine/palmatine) in decoction slices of CREA, CREB, and CREC.

Major component	CREA	CREB	CREC
Berberine (BBR)	4.31 ± 0.30%	4.31 ± 0.31%	4.04 ± 0.39%
Jatrorrhizine (JAT)	1.17 ± 0.02%	1.10 ± 0.02%^a^	0.89 ± 0.05%^b, c^
Coptisine (COP)	1.63 ± 0.04%	1.52 ± 0.02%^a^	1.05 ± 0.04%^b, c^
Palmatine (PAL)	1.26 ± 0.02%	1.26 ± 0.02%	1.26 ± 0.02%
JAT+COP+PAL	4.05 ± 0.06%	3.88 ± 0.04%^a^	3.20 ± 0.10%^b, c^

Notes: Data are presented as the mean ± standard deviation (SD); ^a^significantly different (*p* < 0.05) from CREA; ^b^significantly different (*p* < 0.001) from CREA; ^c^significantly different (*p* < 0.001) from CREB.

### Microbiota-Mediated Metabolism of BBR/JAT/COP/PAL in CREA/CREB/CREC in Rat/Mice Intestinal Contents

**FIGURE 1:**
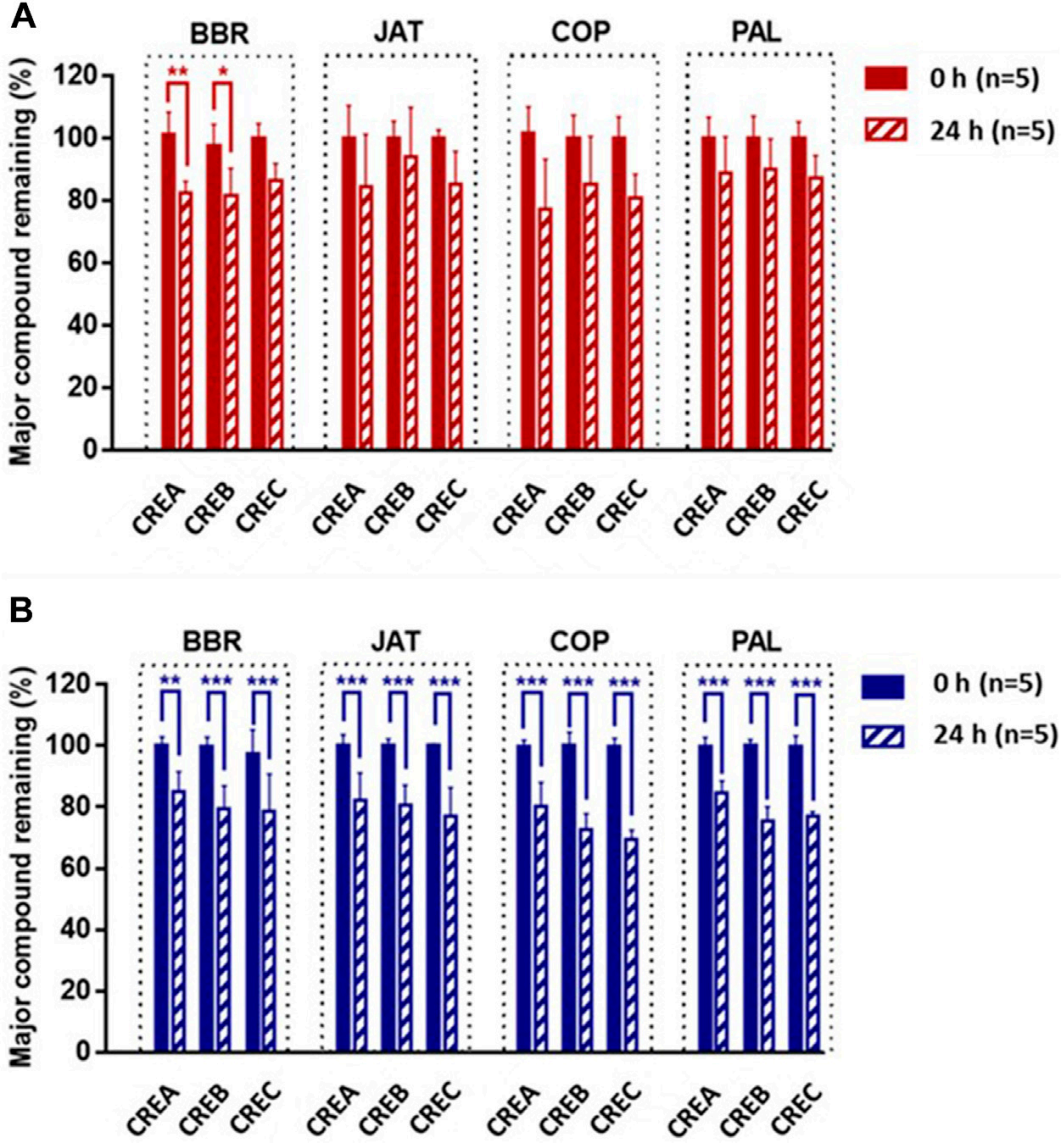
Percentage of BBR, JAT, COP, and PAL in CREA, CREB, and CREC remaining after incubation with Sprague-Dawley rat **(A)** and C57BL/6 mouse **(B)** intestinal content suspensions for 24 h (*n* = 5 per group). Data are presented as the mean ± standard deviation (SD). *Significantly different (*p* < 0.05) from 0 h; **significantly different (*p* < 0.01) from 0 h; ***significantly different (*p* < 0.001) from 0 h.

As shown in Figure 1A, following the incubation of CREA/CREB/CREC with rat intestinal content suspensions for 24 h, BBR was the only component (among the four active compounds) resulting in significant degradations in CREA and CREB, at 82.54 ± 3.58 and 81.41 ± 8.99%, respectively. Compared with the results obtained with rat intestinal contents, all four markers in CREA/CREB/CREC were significantly degraded in C57BL/6 mice after 24 h (Figure 1B). Based on the degradation of BBR and changes in the activity of different CRE components in gut microbiota, CREC was suggested to differ from CREA and CREB.

### Selection of Bacterial Strains May Differentiate the Degradation of CREA/CREB/CREC

#### MIC of BBR/JAT/COP/PAL, CREA/CREB/CREC and Their Mixture (Mixture A/B/C)

MIC is the lowest concentration of a compound that prevents the visible growth of a bacterium. The MICs of standard BBR, JAT, COP, and PAL compounds were individually tested using the broth dilution method with nine common gut bacterial strains, including *E. coli*, *K. pneumoniae*, *E. cloacae*, *E. faecalis*, *S. aureus*, *B. fragilis*, *C. perfringens*, *B. bifidum*, and *L. fermentum* (Table 2). Among the selected bacterial species, the MIC values of the four compounds were greater than or equal to 500 μM, indicating limited inhibitory effects on *E. coli*, *K. pneumoniae*, *E. cloacae*, *E. faecalis*, *S. aureus*, and *B. fragilis*. Conversely, MIC values of BBR/JAT/COP/PAL were within the range 62.5–250 and 31.25–62.5 μM on *C. perfringens* and *B. bifidum*, respectively, demonstrating moderate inhibitory activities. Moreover, only BBR and COP exerted inhibitory effects on *L. fermentum* at an MIC of 250 μM. To verify the MIC of different CREs on the nine selected bacterial strains, standard BBR and CREA/CREB/CREC were incubated at various concentrations. BBR concentrations (2.5320 μM, two-fold serial dilution) in each CRE were standardized and used to determine the MIC values. As shown in Table 2, the inhibitory activity of CREA/CREB/CREC (BBR in CREs ranged from 5 to 160 μM) on *S. aureus*, *B. fragilis*, *C. perfringens*, *B. bifidum*, and *L. fermentum* was stronger than that of the pure BBR compound. When the concentration of BBR in CREA/CREB/CREC was one-eighth of the BBR standard concentration, it was still able to inhibit bacterial growth. Since the components in CREs are complex, the major constituents in CREs might synergize to prevent bacterial growth. Based on the inhibitory activities of CREA/CREB/CREC against the five bacterial strains noted above, we prepared physical mixtures of CREA/CREB/CREC (Mixture A/B/C) by adding the standard compounds of BBR, JAT, COP, and PAL according to the content amount with a consistent ratio in CREA/CREB/CREC to perform parallel MIC determinations with each CR water extract. Three bacteria (*C. perfringens*, *B. bifidum,* and *L. fermentum*) were potently inhibited with a MIC ranging from 0.08 mg/ml (BBR at 10 μM) to 1.28 mg/ml (BBR at 160 μM) of the CREs. Compared with the CREA/CREB/CREC and their Mixture A/B/C, Mixture A/B/C presented weaker inhibition of bacterial growth, with MIC values two- to four-fold higher than that of CREA/CREB/CREC on the probiotic strains (*B. bifidum* and *L. fermentum*). In addition, CREA/CREB/CREC exerted the strongest inhibitory activity on *C. perfringens* among the three bacteria, with a MIC of 0.08–0.16 mg/ml (contained BBR at 10–20 μM).

**TABLE 2:** MIC (μM) of BBR/JAT/COP/PAL standard compound, CREA/CREB/CREC, and their physical mixture (Mixture A/B/C), and on the selected bacterial strains.

		Bacterial strain								
		*E. coli*	*K. pneumoniae*	*E. cloacae*	*E. faecalis*	*S. aureus*	*B. fragilis*	*C. perfringens*	*B. bifidum*	*L. fermentum*
Pure compound (tested conc.: 0.975–1,000 μM)	BBR	>1,000	>1,000	>1,000	>1,000	500–1,000	500	62.5–125	31.25–62.5	250
	JAT	>1,000	>1,000	1,000	>1,000	>1,000	>1,000	250	31.25–62.5	500–1,000
	COP	1,000	>1,000	>1,000	>1,000	>1,000	500	62.5	31.25–62.5	250
	PAL	>1,000	>1,000	>1,000	>1,000	>1,000	>1,000	125	31.25–62.5	500–1,000
BBR *vs.* CRE (tested conc. contained BBR: 2.5–320 μM)	BBR	>320	>320	>320	>320	320	320	80	40	>320
	BBR in CREA	>320	>320	>320	320	160	80–160	10	5	40
	BBR in CREB	>320	>320	>320	320	160	80–160	10	5	20–40
	BBR in CREC	>320	>320	>320	320	160	80–160	10–20	5	20
Compound mixture *vs.* CRE (tested conc. contained BBR: 2.5–320 μM)	Mixture A					>320	320	80	80–160	160
	CREA					160–320	80–160	10	80	40–80
	Mixture B					>320	320	80	160	160
	CREB					160–320	80–160	10–20	80	40–80
	Mixture C					>320	320	80	160	160
	CREC					160–320	80–160	20	80	40–80

Notes: E. coli, K. pneumoniae, E. cloacae, E. faecalis, S. aureus, B. fragilis, *and* C. perfringens *are pathogenic bacteria;* B. bifidum *and* L. fermentum *are probiotic strains*.

#### Time-Kill Kinetics and ATP Production Assays of CREA/CREB/CREC With *C. perfringens*

**FIGURE 2:**
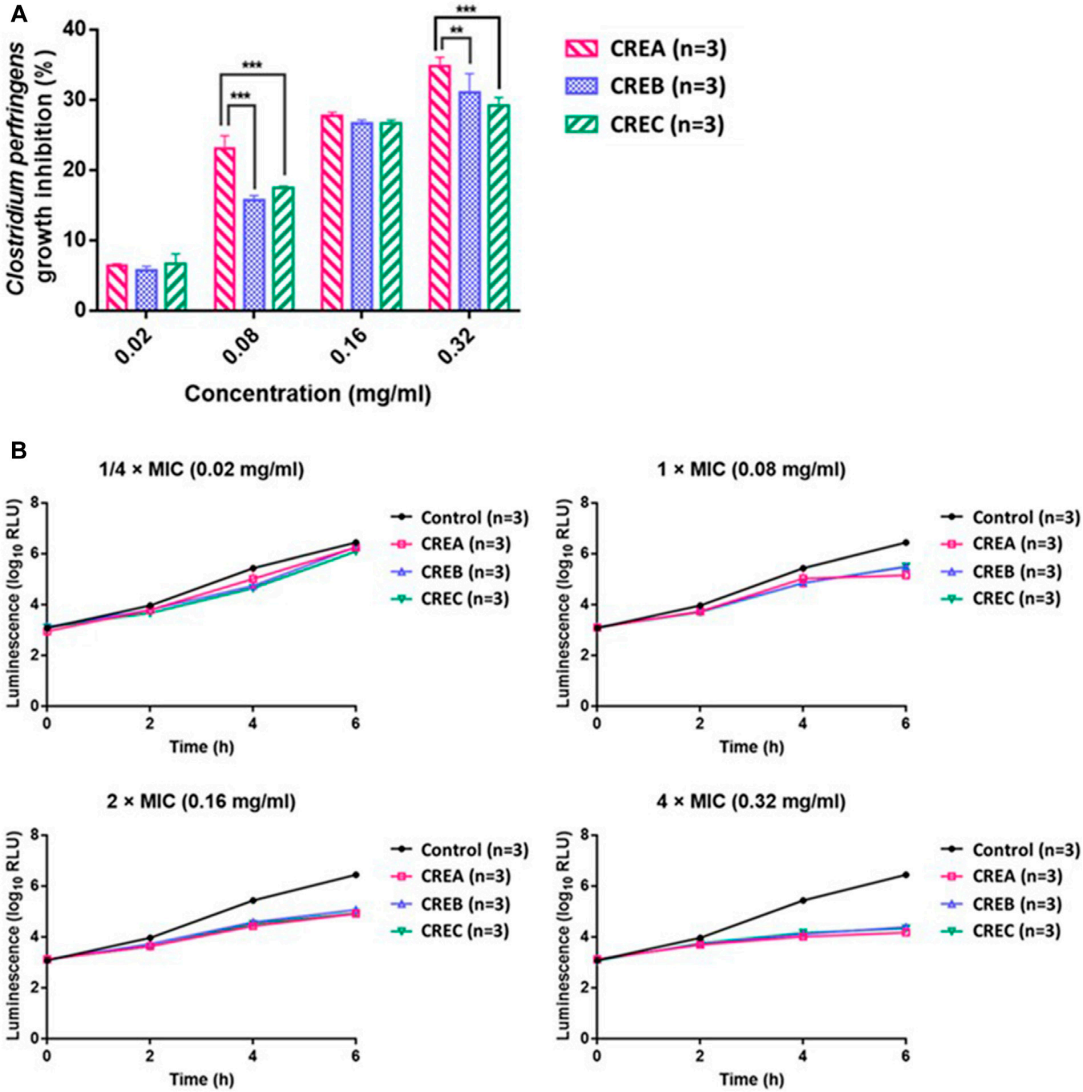
Inhibitory activity (%) of CREA, CREB, and CREC at different concentrations (0.02, 0.08, 0.16, and 0.32 mg/ml) on *C. perfringens* growth after 6 h incubation **(A)**. ATP production by *C. perfringens* during incubation with CREA, CREB, and CREC at various concentrations **(B)**. Data are presented as the mean ± SD. **Significantly different (*p* < 0.01) from CREA; ***significantly different (*p* < 0.001) from CREA (*n* = 3 per group).

Based on the MIC of CREA/CREB/CREC on the selected bacterial strains, 0.08 mg/ml of each CRE was used as 1 × MIC to conduct the time-kill kinetics and ATP production assays on the pathogenic bacteria (*C. perfringens*). Time-kill kinetics revealed the anti-bacterial activities of CREA/CREB/CREC against *C. perfringens* at different concentrations over time. Furthermore, the area under the curve (AUC) of the time-kill curves was applied to calculate the inhibition percentage of bacterial growth by CREA/CREB/CREC as follows: bacterial growth inhibition rate (%) = (AUC _Control_ – AUC _CRE incubation concentration_)/AUC _Control_ * 100. As shown in Figure 2A, after 6 h incubation with *C. perfringens*, the inhibition rate increased with increasing CRE concentrations. In addition, compared to CREB/CREC, CREA significantly inhibited bacterial growth at 0.08 and 0.32 mg/ml, with inhibition rates of 23.09 ± 1.79 and 34.74 ± 1.31%, respectively (*p* < 0.001). There were no significant differences with CREB and CREC at any incubation concentration. Since ATP production is an essential marker of viable cell metabolism in the microbiome, we monitored ATP production while assessing time-kill kinetics at specific time points in the presence of CREA/CREB/CREC at four concentrations in the *C. perfringens* suspensions. As shown in Figure 2B, ATP production started to decrease at a 1 × MIC (0.08 mg/ml) of CREA/CREB/CREC compared to the untreated control group. As the CRE concentrations (2 × MIC and 4 × MIC) increased, ATP production reduced markedly.

### Modulation of Blood Glucose Level and Gut Microbiota Composition After CREA/CREB/CREC Treatment in d*b/db* Mice

#### Body Weight

After 2 weeks CRE treatment, the body weight of mice showed no significant difference between the diabetic control group (39.4 ± 1.3 g) and the three CREs treatment groups (39.2 ± 2.7, 41.5 ± 1.0, and 39.8 ± 2.2 g for CREA, CREB, and CREC, respectively).

#### Blood Glucose Levels in ITT and ipGTT

Blood glucose values obtained from the ITT/ipGTT were plotted against the sampling time. Insulin resistance was compared between treatment groups using the AUC_0→120 min_ of these profiles. As expected, the glucose levels in the diabetic control group were significantly higher than that in the WT group. As shown in Table 3, among the three groups of CRE-treated d*b/db* mice, blood glucose in the CREA treatment group was significantly decreased in comparison to that in the diabetic control group. The results of the ITT and ipGTT in the CREA group reduced from 3303.9 ± 134.3 to 2779.5 ± 521.0 mmol/L*min and from 3708.0 ± 110.7 to 3348.3 ± 78.2 mmol/L*min, respectively. No obvious differences in ITT or ipGTT were observed among the three CRE treatment groups.

**TABLE 3:** Comparisons of ITT (AUC_0→120 min_), ipGTT (AUC_0→120 min_), serum insulin concentration, and HOMA-IR level in different treatment groups of mice.

Test	WT (*n* = 5)	db (*n* = 5)	CREA (*n* = 5)	CREB (*n* = 5)	CREC (*n* = 5)
ITT (AUC_0→120 min_, mmol/L*min)	777.5 ± 42.7	3303.9 ± 134.3^a^	2779.5 ± 521.0^a**,** c^	2956.5 ± 176.5^a^	2870.1 ± 196.9^a^
ipGTT (AUC_0→120 min_, mmol/L*min)	909.3 ± 61.5	3708.0 ± 110.7^a^	3348.3 ± 78.2^a**,** c^	3579.9 ± 128.0^a^	3484.2 ± 339.6^a^
Insulin concentration (mIU/L)	17.0 ± 3.0	259.0 ± 38.1^a^	220.7 ± 58.2^a^	258.4 ± 40.7^a^	231.2 ± 63.6^a^
HOMA-IR	4.0 ± 1.0	222.4 ± 71.0^a^	157.4 ± 74.1^b^	162.2 ± 63.0^b^	160.5 ± 40.9^b^

Notes: Data are presented as the mean ± SD; ^b^significantly different (*p* < 0.01) from WT; ^a^significantly different (*p* < 0.001) from WT; ^c^significantly different (*p* < 0.05) from db.

#### Serum Insulin Concentration, HOMA-IR Level

As shown in Table 3, fasting insulin concentration and HOMA-IR level of diabetic control mice were significantly higher than that in WT group. Although there were no differences in the fasting serum insulin and HOMA-IR levels in the CRE treatment groups compared with the diabetic control group, following 14-days’ treatment with CREA, the insulin concentration (CREA at 220.7 ± 58.2 mIU/L *versus* CREB at 258.4 ± 40.7 mIU/L and CREC at 231.2 ± 63.6 mIU/L) and the HOMA-IR levels (CREA at 157.4 ± 74.1 *versus* CREB at 162.2 ± 63.0 and CREC at 160.5 ± 40.9) tended to decrease in the d*b/db* mice compared to the diabetic control mice.

#### Changes in Gut Microbiota Between CRE Treatment Groups in d*b/db* Mice

**FIGURE 3:**
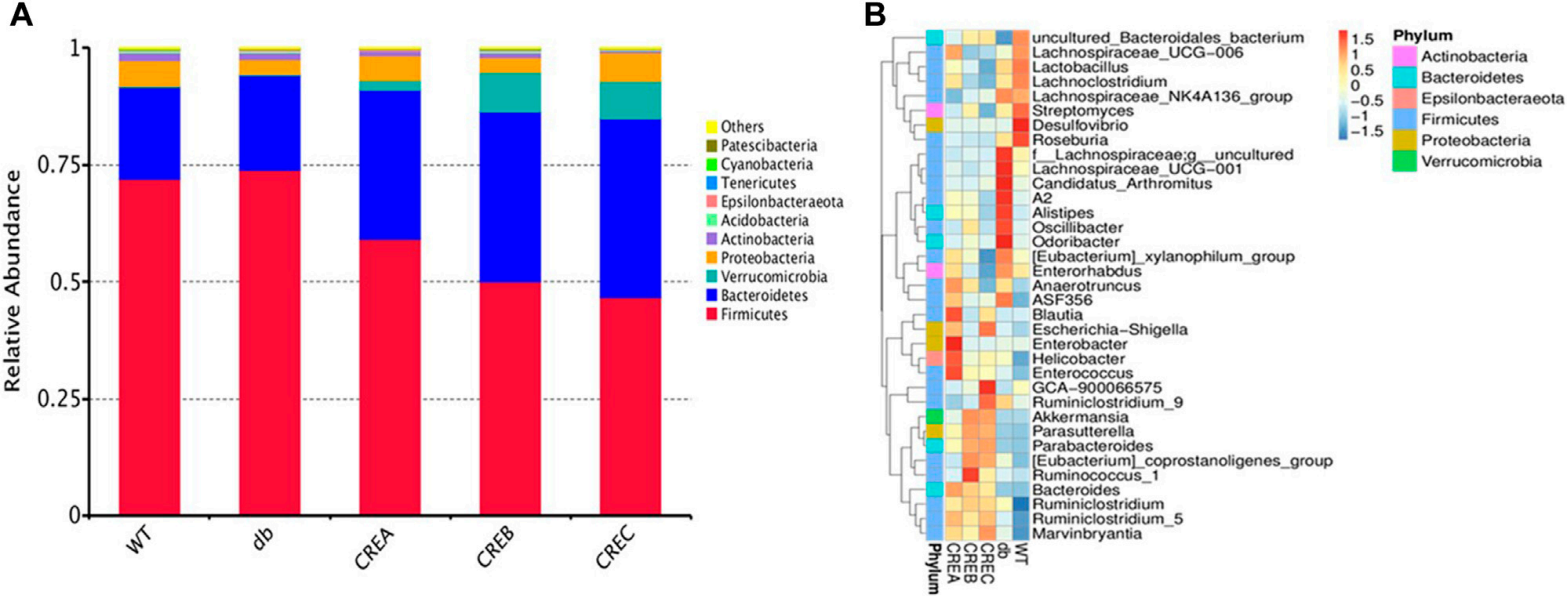
Relative abundance heatmap of intestinal microflora of mice. Top 10 relative bacterial abundance at the phylum level **(A)** and top 35 at the genus level **(B)** among the WT, db, CREA, CREB, and CREC groups (*n* = 5 per group).

Twenty-five fecal samples from mice (five treatment groups with one sample per mouse and five samples per group) with 1,245,075 effective reads (an average of 49,803 ± 4,929 reads per sample) were obtained *via* 16S rRNA amplicon sequencing with QIIME quality selection. As shown in Figure 3A, the abundances of *Firmicutes*, *Bacteroidetes*, *Verrucomicrobia*, *Proteobacteria*, and *Actinobacteria* were highest at the phylum level, and among these, *Firmicutes* was the dominant phylum in all treatment groups. In addition, the ratio of *Firmicutes* to *Bacteroidetes* was significantly decreased after treatment with CREA/CREB/CREC. Furthermore, the abundance of the top 35 genera between the different treatment groups is displayed in Figure 3B. Accordingly, intestinal microbes, such as *Alistipes*, *Oscillibacter*, *Odoribacter*, *Lachnospiraceae UCG−001*, *Candidatus arthromitus*, and *A2,* were more abundant in the diabetic control group (db group) than in the WT group, while their abundance was significantly decreased following CREA/CREB/CREC treatments. *Blautia*, *Enterobacter*, *Helicobacter*, and *Enterococcus* were abundant only in the CREA treatment group. The abundances of *Akkermansia*, *Parasutterella*, *Parabacteroides*, and [*Eubacterium*] *coprostanoligenes group* were increased in both the CREB and CREC groups.

**FIGURE 4:**
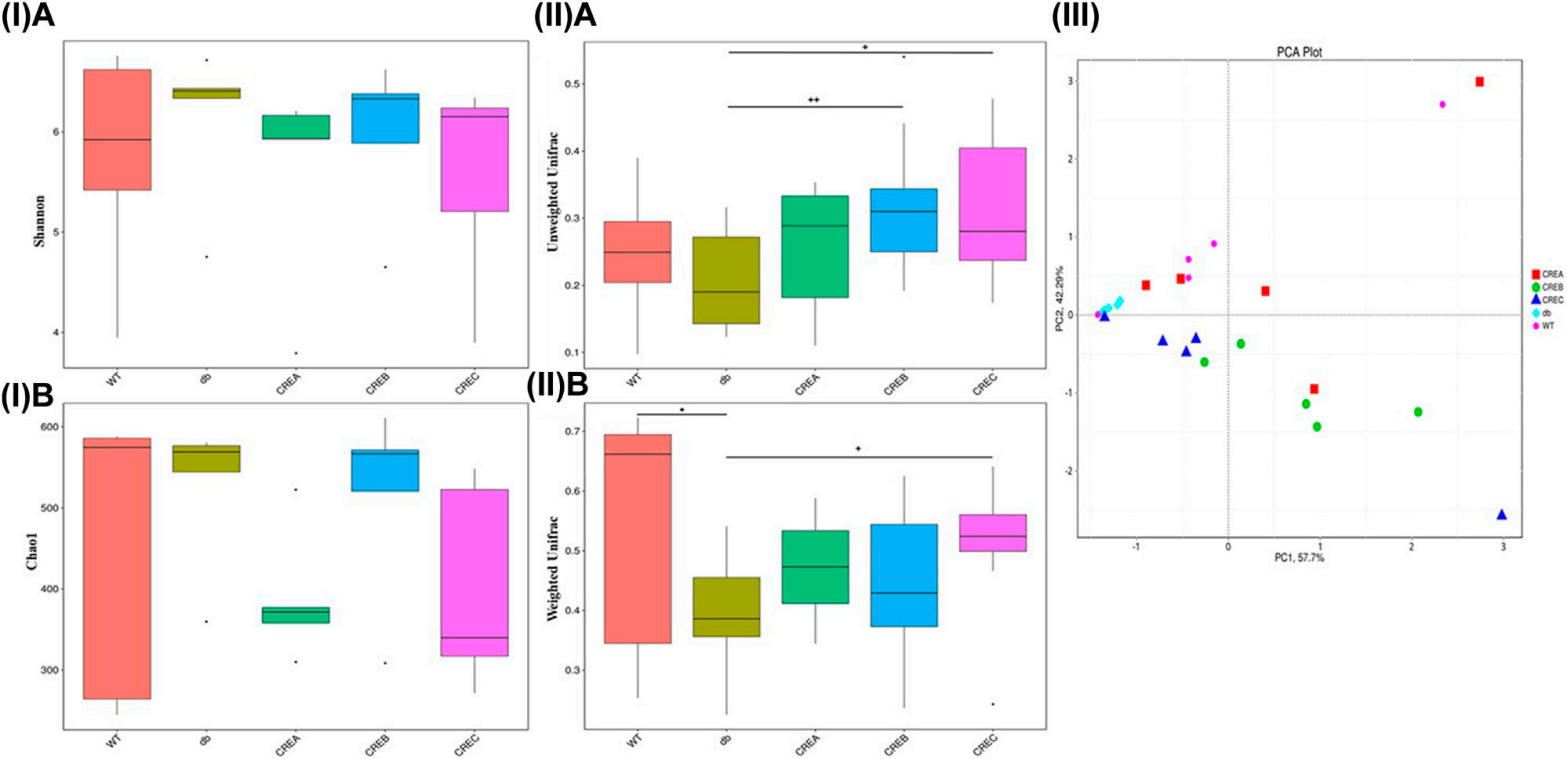
Diversity/richness of gut microbiota and principal component analysis (PCA) analysis among the WT, db, CREA, CREB, and CREC groups (*n* = 5 per group) of mice. (I) Comparisons based on Shannon diversity index **(A)** and Chao1 diversity index **(B)**. (II) Comparisons in the Beta diversity index based on unweighted Unifrac **(A)** and weighted Unifrac **(B)** metrics. (III) PCA analysis based on OTUs clustering. Each data point represented an individual sample. *Significantly different (*p* < 0.05) from WT; +significantly different (*p* < 0.05) from db; ++significantly different (*p* < 0.01) from db.

In addition to analyses of bacterial abundance, boxplots based on the Shannon and Chao1 indices shown in Figure 4 revealed the diversity and richness of the gut microbiota in each treatment group. As indicated in Figures 4A,B, i, the CREA group tended to decrease gut microbiota diversity compared to the diabetic control group based on both Shannon and Chao1 diversity indices. Additionally, the unweighted and weighted Unifrac distances of all treatment groups were compared based on beta diversity. As indicated in Figure 4ii, compared to the diabetic control group, the gut microflora diversity was significantly higher in the CREB and CREC groups according to the unweighted UniFrac index (Figure 4Aii). Regarding comparisons of the weighted Unifrac matrix between groups, the WT group presented significantly greater diversity of gut microflora compared with the diabetic control group. Conversely, in mice treated with CREC, the diversity of microbiota was significantly increased compared to that of the diabetic control group (Figure 4Bii). To analyze differences in microbial community clustering at the genus level, two-dimensional principal component analysis (PCA), as shown in Figure 4iii, was used to illustrate the similarities among different treatment groups. The PCA plot suggested that bacterial communities were altered following CRE treatment in d*b/db* mice. Notably, the intestinal microbiota composition in CREA was most similar to that in the WT group. CREB and CREC treatments revealed differences in gut microbiota modulation compared to the WT control, diabetic control, and CREA treatment groups.

**FIGURE 5:**
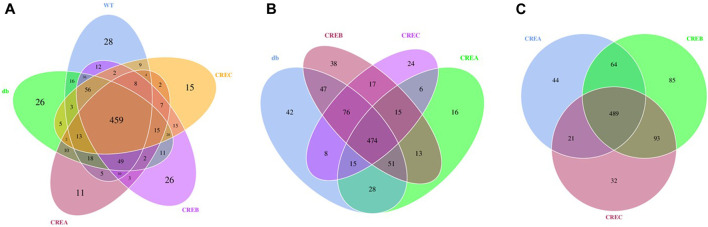
Venn diagram based on comparisons of overlapping and exclusive OTUs of different treatment groups of mice. **(A)** Comparisons among the WT, db, CREA, CREB, and CREC group. **(B)** Comparisons among the db, CREA, CREB, and CREC groups. **(C)** Comparisons among the CREA, CREB, and CREC groups. *n* = 5 per group.

A Venn diagram was used to analyze OTUs clustering among the different treatment groups. Each treatment group, including CRE-treated and WT/db groups, contained unique OTUs in the gut microbial communities (Figure 5A). Comparing the three CRE treatment groups with the diabetic control group, the CREA group presented the least OTUs clustering, with only 16 exclusive OTUs (Figure 5B), whereas CREB and CREC had 38 and 24 exclusive OTUs, respectively. Comparison of OTUs among the three CRE treatment groups suggested that the CREB group presented the highest number of exclusive OTUs clustering (85 OTUs), whereas CREA and CREC groups contained 44 and 32 exclusive OTUs clustering, respectively (Figure 5C).

#### Correlation Analyses of Gut Microbiota Alteration and Glucose Lowering Activity Among Different CRE Treatments in d*b/db* Mice

**FIGURE 6:**
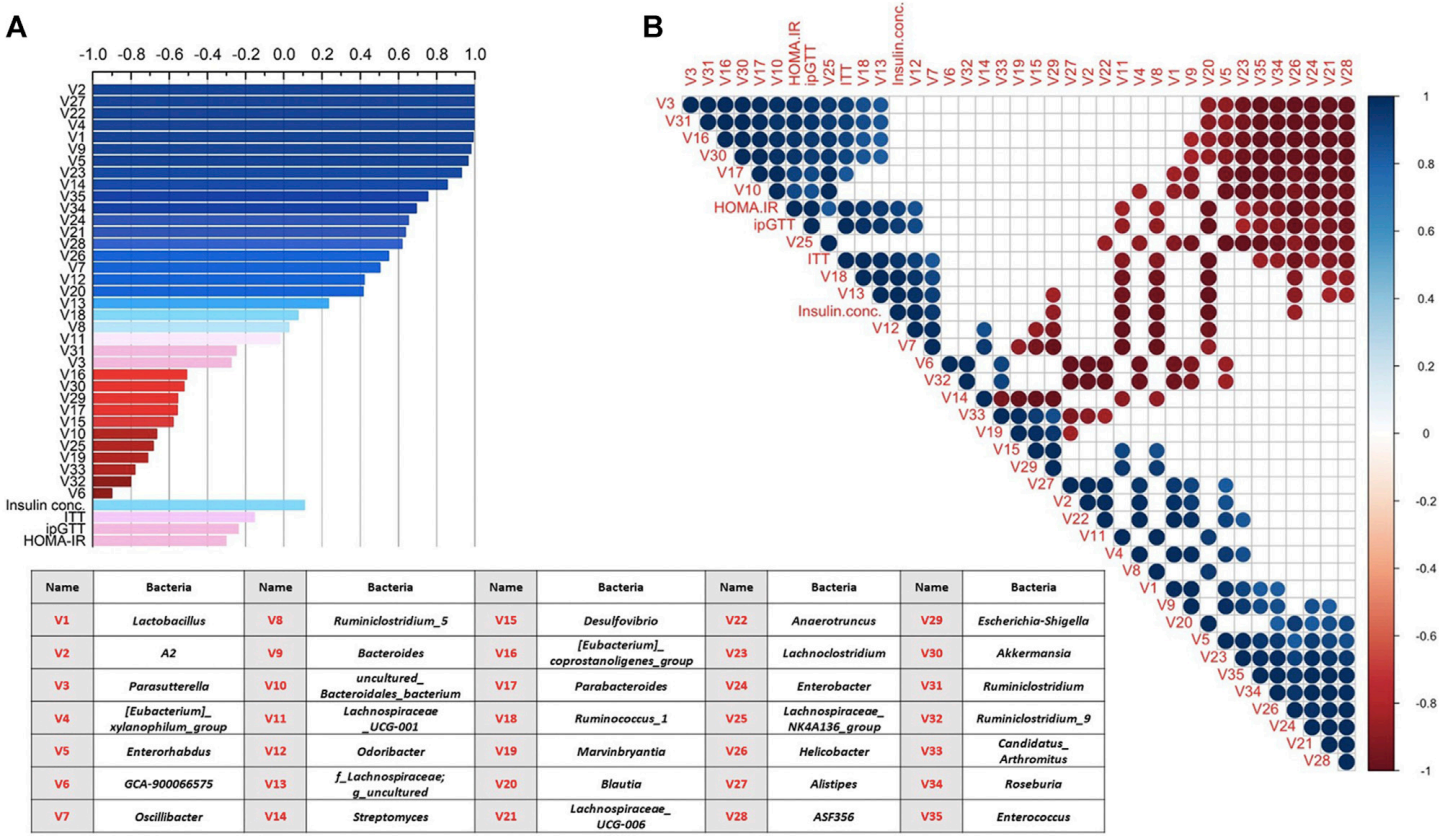
Correlation analyses of gut microbiota alteration (top 35 relative bacterial abundance) and glucose lowering activity among different CRE treatments in d*b/db* mice. **(A)** Correlation analysis between total amount of BBR/JAT/COP/PAL in the three CREs with their alterations on bacterial abundance/ITT/ipGTT/serum insulin concentration/HOMA-IR level. **(B)** Correlation analysis among alterations on bacterial abundance/ITT/ipGTT/serum insulin concentration/HOMA-IR level after the three CRE treatments. Blue and red represented positive and negative correlation, respectively (*p* < 0.05). Blank represented insignificant correlation. V1 to V35 represented 35 bacteria illustrated in the table.

Pearson correlation analyses between the total amount of BBR/JAT/COP/PAL in the three CREs and the alteration of top 35 bacterial relative abundance, ITT, ipGTT, fasting insulin concentration, and HOMA-IR level were conducted and showed in Figure 6A. It was noted that the increased total amount of BBR/JAT/COP/PAL was positively correlated with the abundance of bacteria such as *A2*, *Alistipes*, *Anaerotruncus*, *Lactobacillus*, and *Bacteroides*, while negatively correlated with *Ruminiclostridium_9*, *Candidatus_arthromitus*, *Marvinbryantia*, etc. Moreover, insulin resistant assessments including ITT, ipGTT and HOMA-IR level were negatively correlated with the increased total amount of BBR/JAT/COP/PAL in CREs, further demonstrating that CREA with the highest amount of BBR/JAT/COP/PAL had the most potent glucose lowering effect in d*b/db* mice.

In addition, Pearson correlation analyses were also conducted among alterations on bacterial abundance/ITT/ipGTT/serum insulin concentration/HOMA-IR level after the three CRE treatments as shown in Figure 6B. It was indicated that bacteria showed mutual influence on their relative abundance with positive/negative correlation after the different CRE treatments. The abundance of *Parasutterella*, *Ruminiclostridium*, *Akkermansia*, and *Parabacteroides* were negatively correlated with that of *Lachnospiraceae_UCG-006*, *Enterobacter*, *Helicobacter*, *Roseburia*, *Enterococcus*, *Lachnoclostridium*, and *Enterorhabdus*. Furthermore, ITT, ipGTT, insulin concentration and HOMA-IR level were positively correlated with the relative abundance of *Odoribacter* and *Ruminococcus_1* and negatively correlated with that of *Ruminiclostridium_5*, *Lachnospiraceae_UCG-001*, *Blautia*, and *Helicobacter*.

## Discussion

CR, a well-known herb in traditional Chinese medicine, contains four major active constituents (BBR, JAT, COP, and PAL) and a further 125 identified components (Wang et al., 2019). CR has been shown to have potent impact on T2D and strongly affect the modulation of gut microbiota. Previous findings indicated that CREs could enhance insulin sensitivity by inhibiting the pancreatic lipid peroxidation, upregulating the expression of *p*-AMPK-A protein, increasing the glucose uptake in preadipocytes, and reducing the inflammatory cytokine levels in rats (Song et al., 1995; Li et al., 2010; Wang H. et al., 2014; Xu et al., 2020). To our knowledge, this is the first study to investigate the effects of different CREs on the modulation of gut microbiota and blood glucose control in diabetic d*b/db* mice. The results support the feasibility of using gut microbiota modulations to differentiate the quality of different CR herbal extracts.

Different levels of BBR, JAT, COP, and PAL in the three CREs, along with their different stabilities in the GI contents of rats/mice, led us to screen their potential differences in the regulation of microbiota. Nine common GI bacterial strains were selected for MIC determinations. Previous studies have shown that BBR and PAL exert inhibitory effects on the growth of *C. perfringens* and *B. bifidum*, but only weak inhibitory effects of *E. coli* (Chae et al., 1999); this was consistent with the findings of the present study. The results of the time-kill kinetics study and ATP production assays on *C. perfringens* indicated that CREA exerted the greatest inhibitory effects on this pathogenic bacterium, followed by CREB and CREC. The ranking of CREs was consistent with the ranking of blood glucose-lowering effects observed in d*b/db* mice.

As indicated by previous findings, CRE at 1.64 g/kg led to 25% death of mice and the median acute oral lethal dose of CRE was 2.95 g/kg in mice (Ma et al., 2010). The dose of CREs in our current investigation was only 200 mg/kg, which is equivalent to the lowest clinical dose (2 g/day/person) (China Pharmacopeia Commission, 2015), and much lower than those lethal doses reported in the previous literature. In addition, long-term administration of BBR at 93.75 mg/kg (9 weeks) has been reported to demonstrate the beneficial effects on the T2D related intestinal mucosal barrier damage (Gong et al., 2017) and COP has also showed the gastric-mucous membrane protection activity (Hirano et al., 2001; Wu et al., 2019). Therefore, the risk of severe adverse effects caused by our selected dose of CREs is considered to be minimal.

CREA was the most potent extract among the three CREs studied, with a strong inhibitory effect on pathogenic bacteria and efficacy in lowering blood glucose activity in d*b/db* mice. However, the significant modulation of *C. perfringens* observed *in vitro* was not observed in the fecal microbial 16S rRNA sequencing in d*b/db* mice, which could be due to differences between the *in vitro* and *in vivo* models. In our *in vitro* incubation study, only isolated and specific bacterial strains were used, without consideration of the mutual influence among different microbes in the GI tract of d*b/db* mice.

Increasing evidence suggests that gut microflora can be modulated in diabetic patients and T2D related animal models. As discussed in a review by Gurung et al., the diversity or richness of gut microbiota in patients with T2D shared little similarities due to the large individual differences. *Bifidobacterium* and *Bacteroides* are commonly regarded as beneficial genera, and result in improved glucose tolerance and insulin resistance in T2D (Munukka et al., 2012; Candela et al., 2016; Yamaguchi et al., 2016; Lippert et al., 2017; Barengolts et al., 2018; Gao et al., 2018). Studies have shown that *Akkermansia*, *Faecalibacterium*, and *Roseburia* are more abundant in healthy control groups than in patients with T2D (Zhang et al., 2013; Candela et al., 2016; Greer et al., 2016; Gao et al., 2018; Salamon et al., 2018; Gurung et al., 2020). Although the relationship between the ratio of *Firmicutes* to *Bacteroidetes* in gut microbiota and diabetic status remains controversial (Gurung et al., 2020), it has been demonstrated to be positively associated with T2D in d*b/db* mice (Geurts et al., 2011). In the present study, we used d*b/db* mice to investigate the effects of different CREs on the modulation of gut microbiota, and we found that *Firmicutes* and *Bacteroidetes* were the dominant phyla in all d*b/db* mice treatment groups, which is consistent with the previous literature (Geurts et al., 2011; Cai et al., 2020). Although studies investigating the modulation of microbiota by CREs in d*b/db* mice are lacking, the anti-diabetic effects of BBR associated with the modulation of gut microbiota have been reported widely in different diabetic animal models, including d*b/db* mice and high-fat diet-fed rats/mice (Habtemariam, 2020).

In response to the different CRE treatments in our study, the ratio of *Firmicutes* to *Bacteroidetes* was significantly decreased compared to that of the diabetic control. Furthermore, the relative abundances of *Alistipes*, *Odoribacter*, and *Oscillibacter* decreased at the genus level following CREA/CREB/CREC treatment, which was not observed in a previous report on BBR (Li et al., 2020). Unlike CREA, CREB/CREC treatment led to an increased abundance of *Akkermansia*, which was consistent with the increased abundance of *Akkermansia* in d*b/db* mice following oral treatment with BBR at 136.5 mg/kg for 11 weeks (Zhang et al., 2019) or 100 mg/kg for 55 days (Li et al., 2020). Comparing the modulation of gut microbiota in d*b/db* mice between CRE treatment in our study and BBR treatment in the study by Li et al. (Li et al., 2020), both treatments decreased the ratio of *Firmicutes* to *Bacteroidetes*, whereas changes in the genus *Lactobacillus* are conflicting. Li et al. revealed that BBR significantly decreased the diversity of microbial communities (Li et al., 2020), while in our study, this enrichment was demonstrated in CREB and CREC treatment groups. Based on the heatmap abundance at the genus level in the current study, CREA/CREB/CREC enriched the relative abundance of *Bacteroides*, which has been recognized as a genus important for glucose tolerance in T2D (Munukka et al., 2012; Zhang et al., 2013; Candela et al., 2016; Yamaguchi et al., 2016; Lippert et al., 2017). Our further correlation analyses also indicated the positive correlation between relative abundance of *Bacteroides* and the total amount of BBR/JAT/COP/PAL in CREs, suggesting the important role of *Bacteroides* in the diabetic treatment. Among the 35 top bacterial abundance, 21 of them were positively correlated with the total amount of BBR/JAT/COP/PAL in CREs. Therefore, the components of CREs, dosage, and dosing frequency, and bio-interindividual diversity in mice may result in differences in terms of gut microbiota alterations.

Although marker bioactive components such as BBR/JAT/COP/PAL have been widely adopted by guidelines as a method of quality control for CREs, such methods are barely able to differentiate CREs from different sources and have no direct indications and medications of their related pharmacological activities. In the current study, we used CREs to demonstrate the important role of gut microbiota in differentiating CREs from different sources. We investigated the effects of gut microbiota from rodents on the major components of CREs and evaluated the effects of major active ingredients (BBR/JAT/COP/PAL) and different CREs on common GI bacteria. Despite marginal differences in the active marker components in the three types of CREs, CREA, the genuine medicinal material, demonstrated a stronger inhibitory effect on GI bacterial growth and on blood glucose compared to CREB and CREC in d*b/db* mice. Since our current study serve as a novel pilot exploration on relationship between microbiota alteration and glucose lowering effect of CRE treatment in d*b/db* mice, future studies on the selected potent CREs such as CREA and its components could be conducted on exploring their related mechanistic pathways including lipid profile and metabolic profile changes.

In summary, the major active components of CREs had synergistic effects on common GI bacteria. CREs with different BBR, JAT, COP, and PAL compositions differed in their time-kill kinetics and ATP production for *C. perfringens*. Among the three CREs studied, CREA demonstrated the greatest inhibitory effect on *C. perfringens* growth. In d*b/db* mice, CREA was found to have the strongest blood glucose-lowering effect, with significant changes in the gut microbiota compared to CREB and CREC.

## Data Availability

Raw sequence data were deposited on the NCBI Sequence Read Archive platform under the BioProject accession number PRJNA715953. The authors declare that all supporting data for the results described in this article are available from the corresponding author upon request, with no restrictions.

## Glossary

ACN: Acetonitrile
AUC: Area under the curve
AUC_0→t_: Area under the curve up to the last measured concentration
BBR: Berberine
*B. bifidum*: *Bifidobacterium bifidum*
*B. fragilis*: *Bacteroides fragilis*
CAF: Caffeine
CMC-Na: Sodium carboxymethyl cellulose
COP: Coptisine
*C. perfringens*: *Clostridium perfringens*
CR: *Coptidis rhizoma*
CREs: *Coptidis rhizoma* extracts
DMSO: Dimethyl sulfoxide
*E. cloacae*: *Enterobacter cloacae*
*E. coli*: *Escherichia coli*
*E. faecalis*: *Enterococcus faecalis*
GAM: Gifu Anaerobic Medium
GI: Gastrointestinal
HOMA-IR: Homeostatic Model Assessment of Insulin Resistance
HPLC: High-performance liquid chromatography
i.p.: Intraperitoneal
ipGTT: Intraperitoneal glucose tolerance test
IS: Internal standard
ITT: Insulin tolerance test
JAT: Jatrorrhizine
*K. pneumoniae*: *Klebsiella pneumoniae*
LC-MS/MS: Liquid chromatography/tandem mass spectrometry
*L. fermentum*: *Lactobacillus fermentum*
MeOH: Methanol
MIC: Minimum inhibitory concentration
MRM: Multiple reaction monitoring
OTUs: Operational taxonomic units
PAL: Palmatine
PCA: Principle component analysis
PCR: Polymerase chain reaction
rRNA: ribosomal RNA
*S. aureus*: *Staphylococcus aureus*
T2D: Type 2 diabetes
