# Supplementary material for: Blood-Glucose-Lowering Effect of Coptidis Rhizoma Extracts From Different Origins via Gut Microbiota Modulation in db/db Mice
Source: Front Pharmacol. 2021 Jun 15;12:684358. doi: 10.3389/fphar.2021.684358 (PMC8239385; doi:10.3389/fphar.2021.684358)
Supplement: Supplementary file 1 [file DataSheet1.docx]

**Legends of Supplementary Figures**

**Supplementary Figure 1.** HPLC/UV spectrum of major active components BBR/JAT/COP/PAL in CREA, CREB and CREC.

**Supplementary Figure 2.** Quantity and integrity of fecal DNA samples in WT, db, CREA, CREB, and CREC group (n=5/group) of mice.


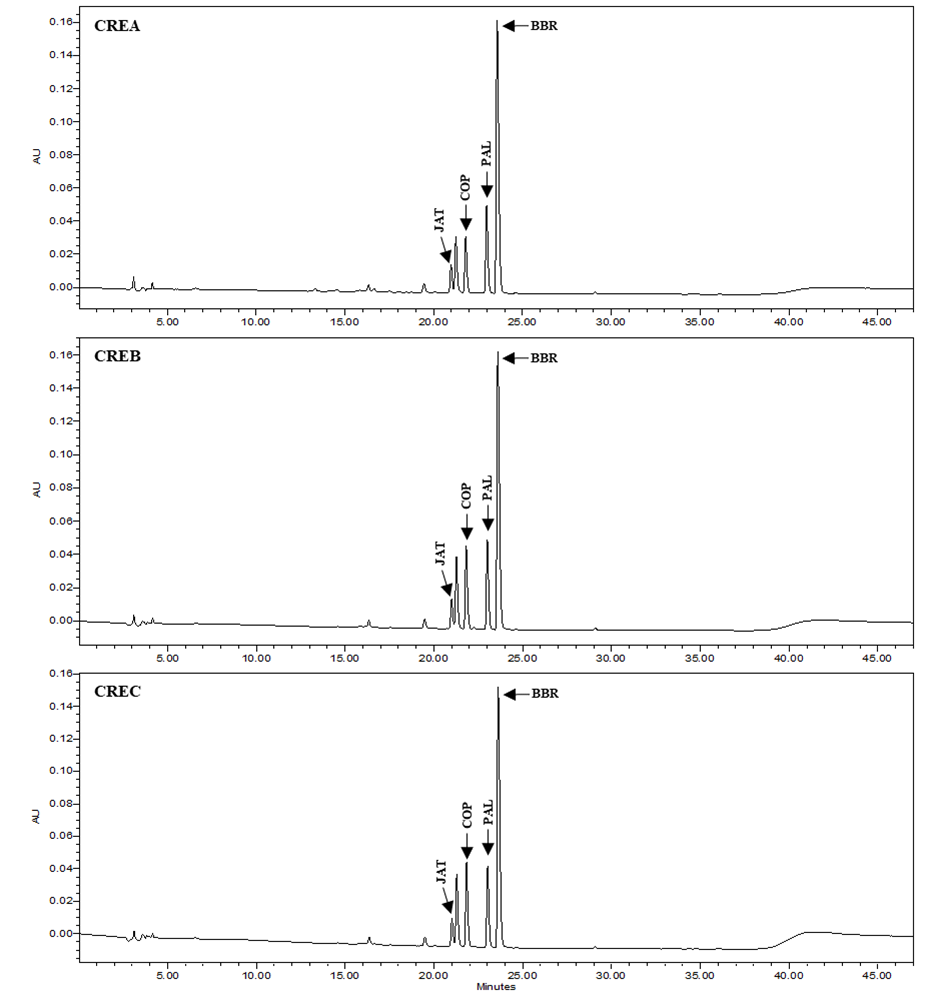


**Supplementary Figure 1.** HPLC/UV spectrum of major active components BBR/JAT/COP/PAL in CREA, CREB and CREC.


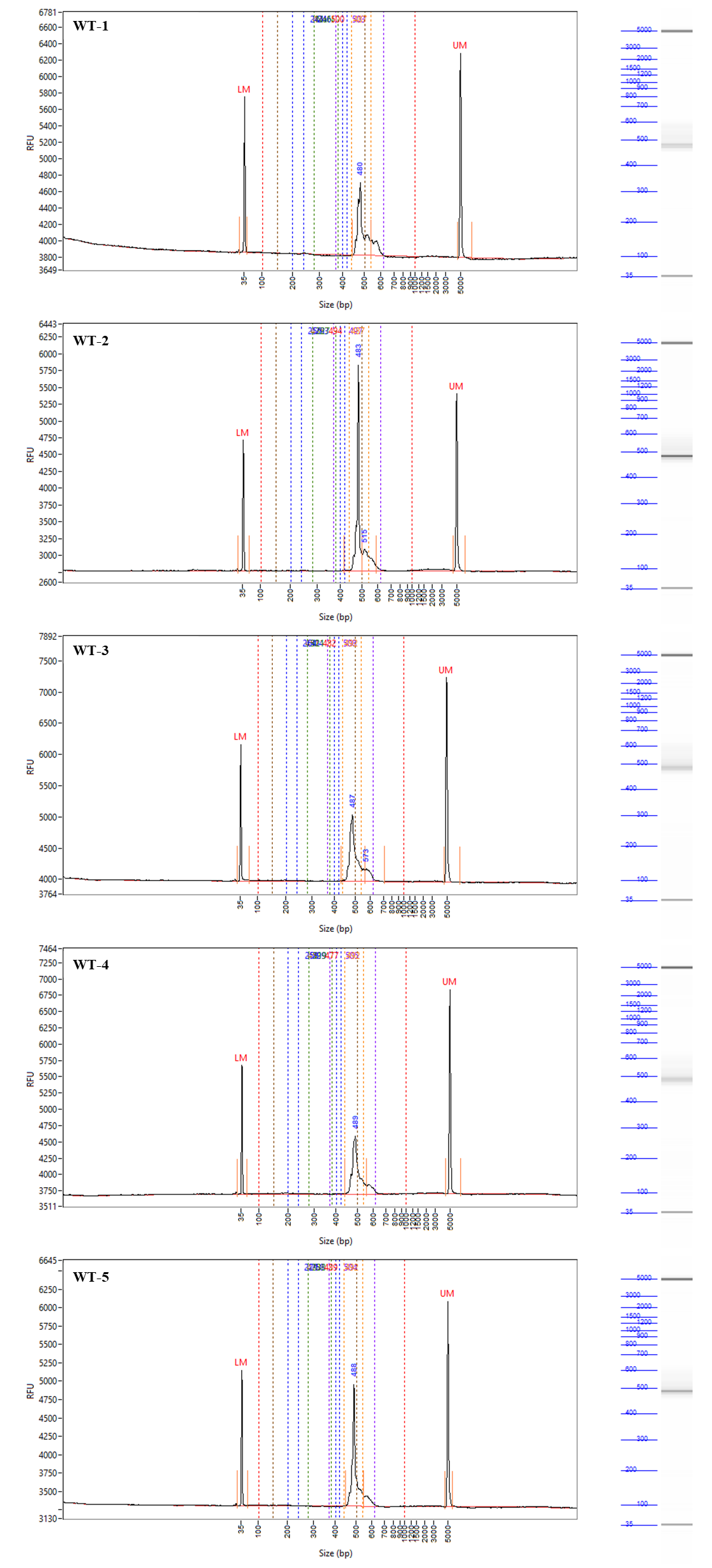

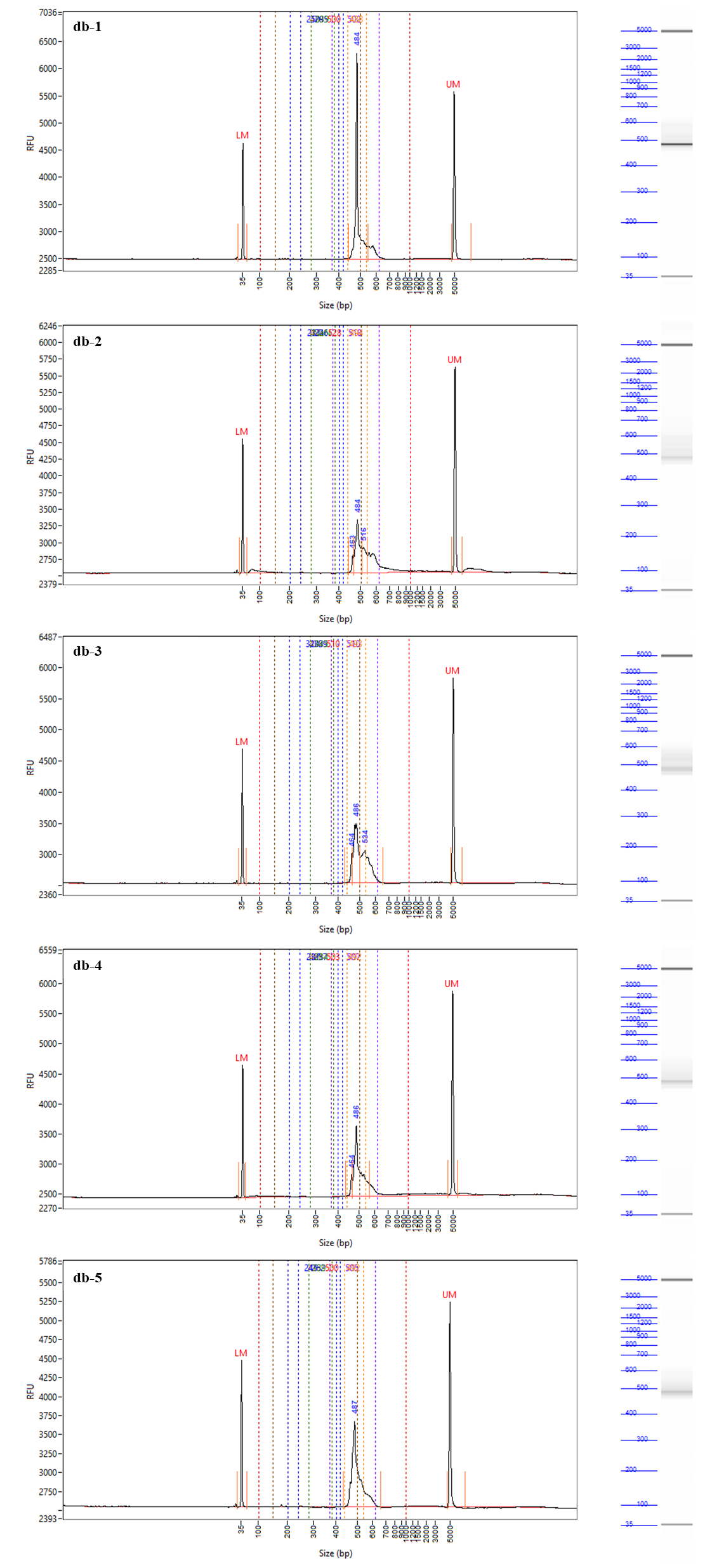


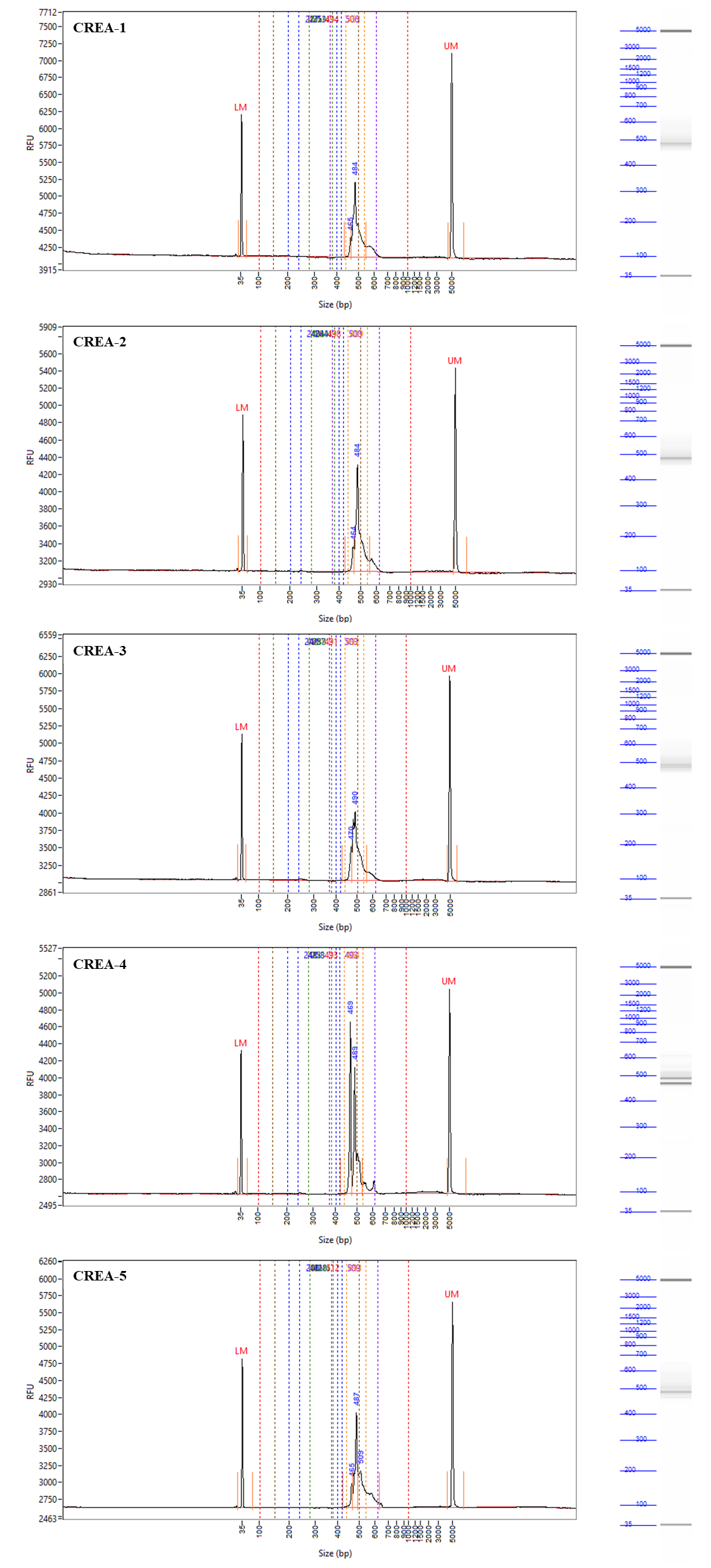


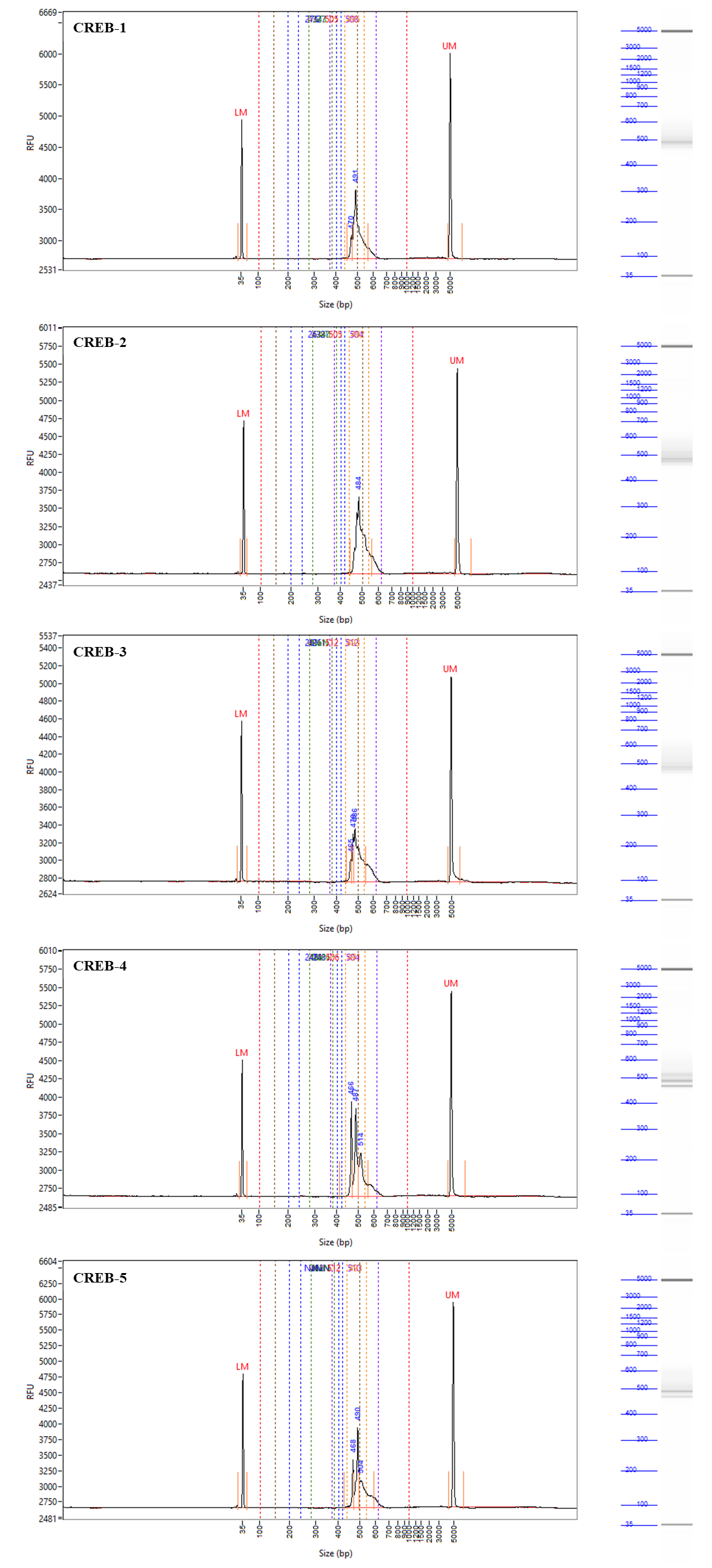


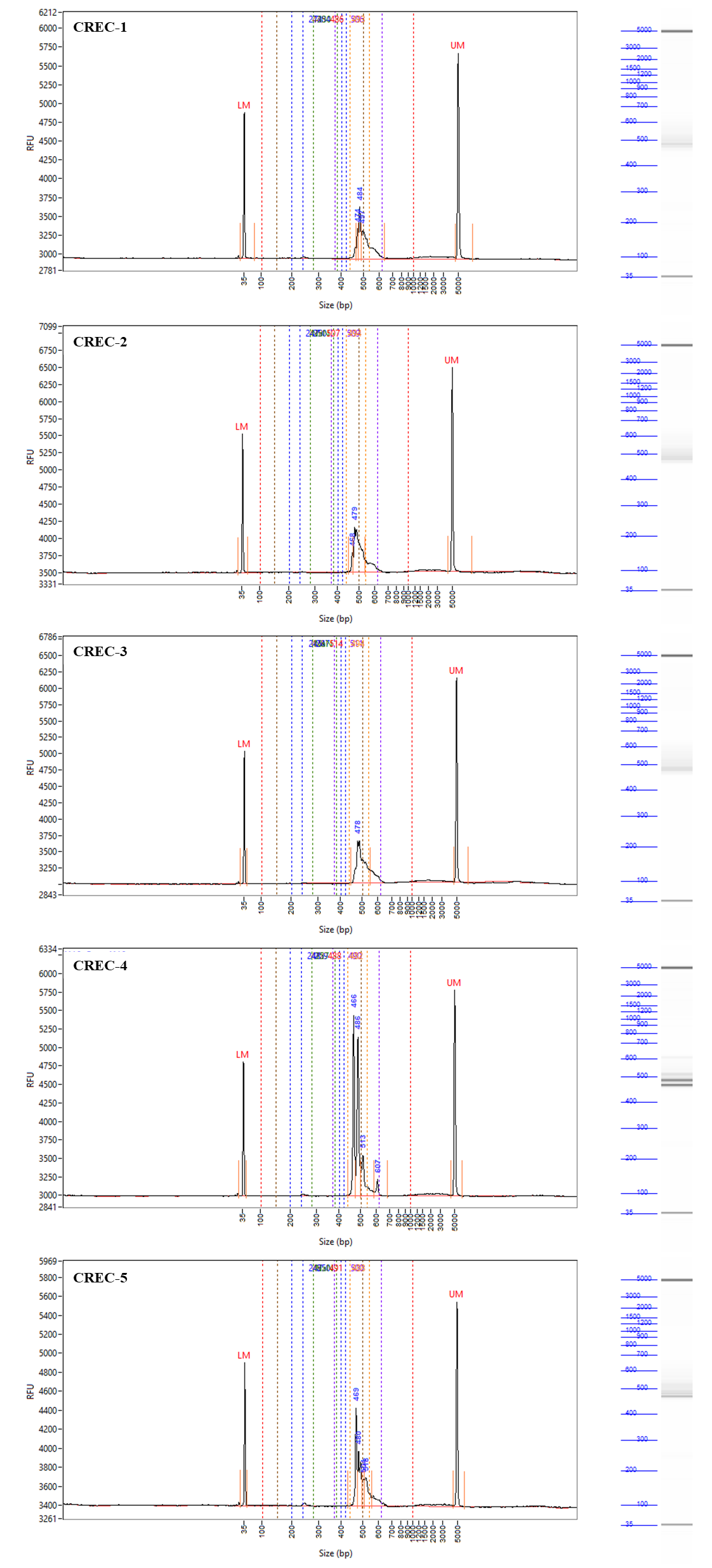


**Supplementary Figure 2.** Quantity and integrity of fecal DNA samples in WT, db, CREA, CREB, and CREC group (n=5/group) of mice.
